# High Glucose Concentration Promotes Vancomycin-Enhanced Biofilm Formation of Vancomycin-Non-Susceptible *Staphylococcus aureus* in Diabetic Mice

**DOI:** 10.1371/journal.pone.0134852

**Published:** 2015-08-05

**Authors:** Chi-Yu Hsu, Jwu-Ching Shu, Mei-Hui Lin, Kowit-Yu Chong, Chien-Cheng Chen, Shu-Min Wen, Yi-Ting Hsieh, Wan-Ting Liao

**Affiliations:** 1 Department of Medical Biotechnology and Laboratory Science, College of Medicine, Chang Gung University, No. 259, Wenhua 1^st^ Road, Guishan, Taoyuan 333, Taiwan; 2 Research Center for Pathogenic Bacteria, Chang Gung University, No. 259, Wenhua 1^st^ Road, Guishan, Taoyuan 333, Taiwan; 3 Department of Laboratory Medicine, Chang Gung Memorial Hospital, No. 5, Fusing St., Guishan, Taoyuan 333, Taiwan; 4 Department of Biotechnology, National Kaohsiung Normal University, No.62, Shenjhong Rd., Kaohsiung 824, Taiwan; Chang-Gung University, TAIWAN

## Abstract

We previously demonstrated that vancomycin treatment increased acquisition of eDNA and enhanced biofilm formation of drug-resistant *Staphylococcus aureus* through a *cidA*-mediated autolysis mechanism. Recently we found that such enhancement became more significant under a higher glucose concentration *in vitro*. We propose that besides improper antibiotic treatment, increased glucose concentration environment in diabetic animals may further enhance biofilm formation of drug-resistant *S*. *aureus*. To address this question, the diabetic mouse model infected by vancomycin-resistant *S*. *aureus* (VRSA) was used under vancomycin treatment. The capacity to form biofilms was evaluated through a catheter-associated biofilm assay. A 10- and 1000-fold increase in biofilm-bound bacterial colony forming units was observed in samples from diabetic mice without and with vancomycin treatment, respectively, compared to healthy mice. By contrast, in the absence of glucose vancomycin reduced propensity to form biofilms *in vitro* through the increased production of proteases and DNases from VRSA. Our study highlights the potentially important role of increased glucose concentration in enhancing biofilm formation in vancomycin-treated diabetic mice infected by drug-resistant *S*. *aureus*.

## Introduction

*Staphylococcus aureus* is one of the leading causes of healthcare-associated and community acquired infections and produces a wide spectrum of diseases, ranging from superficial to life-threatening. The emergence of multidrug-resistant *S*. *aureus* (MDRSA), including methicillin-resistant *S*. *aureus* (MRSA), limits therapeutic options in the clinical environment. Glycopeptides, such as vancomycin, are the antibiotics of choice in treating infections caused by MDRSA. Due to the frequent use of glycopeptides, this pathogen has developed low to intermediate levels of resistance to vancomycin by mutational mechanisms and high levels of resistance by the acquisition of transferrable determinants [1, 2]. A recent study in Taiwan indicated that 2.9% of the MRSA strains isolated were vancomycin-intermediate *S*. *aureus* (VISA) [3].

We studied the role of an antibiotic when it was applied to a resistant strain of bacteria. We previously reported that sub-minimum inhibitory concentrations (sub-MICs) of antibiotics, such as vancomycin, act as environmental stressors and activate the stress response sigma factor, σ^B^, thereby altering the expression of virulence factors in vancomycin-resistant *S*. *aureus* (VRSA) strains [4]. We also showed that these antibiotic treatments enhanced biofilm formation by drug-resistant strains through an increased acquisition of extracellular DNA (eDNA) via a *cidA*-mediated autolysis mechanism [5].

Biofilm formation in medical device-related infections is of increasing importance in the clinical environment because it can provide an extracellular barrier to antimicrobial agents or host immune defenses. Two articles have reviewed mechanisms of staphylococcal biofilm formation as well as biofilm regulation [6, 7]. Formation of a biofilm is initiated by the primary attachment of bacteria onto polymeric surfaces or human matrix proteins via cell wall-associated adhesins, including microbial surface components recognizing adhesive matrix molecules (MSCRAMMs), such as fibronectin-binding proteins and protein A. Maturation is then mediated by the production of extracellular factors, such as the polysaccharide intercellular adhesin (PIA), which is regulated by genes in the *icaRADBC* locus [8]. The molecular basis of staphylococcal biofilm detachment has been reviewed in detail in some recent articles [9–11]. Staphylococcal biofilms are composed of three major components: associated proteins, eDNA, and PIA; PIA is not required for some *S*. *aureus* strains. Thus, biofilm disassembly may be involved in the decreased expression of the above components or the expression of destructive products to degrade the biofilm matrix. The matrix degrading gene products include proteases, DNases, and surfactants, such as phenol-soluble modulins (PSMs).

It has been previously demonstrated that chronic infections or chronic wounds are highly associated with the formation of biofilms [12–14]. Bacterial biofilms have been shown to play a role in perturbing the healing of chronic wounds, especially for patients with diabetes [15, 16]. However, whether the formation of biofilms delays wound healing or the wound environment enhances biofilm formation in patients with diabetes remains unclear. Formation of biofilms is also frequently associated with catheter-related infections, such as central venous catheter (CVC) biofilms in bloodstream infections or urinary catheter biofilms in urinary tract infections, especially in patients with diabetes [17–19]. Thus, biofilm-associated infection has become a major medical problem that results in a significant burden on the health care system.

We recently found that glucose played an important role in enhanced biofilm formation by VRSA upon vancomycin treatment. The higher the concentration of glucose, the more biofilm materials were formed upon vancomycin treatment *in vitro*. Therefore, we studied the interplay among the diabetic environment, bacterial biofilm-forming capacity, and antibiotic treatment, particularly in mice infected with a drug-resistant *S*. *aureus* strain in a diabetic mouse model.

## Materials and Methods

### Bacterial strains and growth conditions

The vancomycin-resistant *S*. *aureus* strain SJC1200 (MIC > 256 μg/ml) was generated by introducing a plasmid (pG1546) carrying a vancomycin resistance operon into strain ATCC 12598 as described previously [5]. Allelic replacement of the *cidA* gene by a spectinomycin cassette (*spc*) was performed by introducing plasmid pMAcid into strain SJC1200 to generate the *cidA* mutant strain SJC1201, according to the method described elsewhere [5]. Vancomycin susceptible strain ATCC 12598 and VISA strain Mu50 were also used in our animal model. All bacterial strains were routinely cultured at 37°C with the specific required antibiotics (Sigma-Aldrich, St. Louis, USA) in BHI broth or on agar plates.

### Static biofilm adherence assay and detection of medium glucose

The static biofilm adherence assay was performed with flat-bottom 96-well polystyrene microtiter plates according to procedures described previously [5, 20]. A single colony of strain SJC1200 from a fresh agar plate was inoculated in BHI broth, incubated overnight at 37°C and diluted to an OD_600_ of 0.05 with fresh BHI media or media supplemented with an additional 0.5% or 1.5% glucose (BHIg or BHIhg, respectively) in the presence or absence of vancomycin (32 μg/ml). Under specified circumstances, a protease inhibitor cocktail (100× dilution; Sigma-Aldrich) was added. Individual wells were filled with 0.2 ml of the diluted cultures (containing approximately 10^7^ CFU/ml), and broth alone served as a control to check for sterility and the non-specific binding of the media. After motionless incubation at 37°C for the indicated time intervals, the plate wells were washed twice with phosphate-buffered saline (PBS, pH 7.2), and the dried adherent cells were stained with 0.1% safranin O. The medium glucose was determined by GOD-PAP method using GLUCOSE liquicolor kit (HUMAN, Germany). The experiments for each condition were performed in triplicate and repeated at least three times.

### Animals and induction of diabetes

Six-week-old male BALB/c mice and WKY/NcrlNarl rats that weighted approximately 150 g were purchased from the National Laboratory Animal Center (Taiwan) and were allowed to acclimatize to the laboratory animal center at Chang Gung University for three days prior to the beginning of the experiments. All of the experimental procedures described were approved by the Chang Gung University Institutional Animal Care and Use Committee (approval No. CGU12-063). Mice or rats fasted for 12 h before diabetes was induced by a single intraperitoneal injection of the pancreatic β-cell toxin streptozotocin (STZ; Sigma-Aldrich) that was freshly dissolved in 0.05 M citrate buffer, pH 4.5 (200 and 300 mg/kg for mice and rats, respectively). The normal animals received injections of citrate buffer only. Blood samples were periodically collected from the tail vein, and glucose levels were determined using a glucometer (Accu-Chek Active, Roche, Switzerland). Animals were considered diabetic if the fasting blood glucose levels were above 250 mg/dl on three consecutive determinations approximately 7 to 10 days after STZ injection.

### *In vivo* model of catheter-associated biofilm formation

For the subcutaneous catheter model, mice were anesthetized with 1.25% 2-2-2 tribromoethanol (0.025 ml/g of body weight; Sigma-Aldrich) and implanted subcutaneously with a 1-cm segment of size Fr-10 silicon catheter in the dorsal area, according to methods described earlier [21]. Infection was initiated immediately after the implantation procedure by injecting approximately 10^3^ CFU of *S*. *aureus* in a total volume of 10 μl of PBS into the lumen of the catheter. The wound was closed with skin staplers after the inoculation of the *S*. *aureus* strains. After 1 h, vancomycin (30 mg/kg/dose), chloramphenicol (25 mg/kg/dose) or control PBS buffer was administered once daily by intraperitoneal injection. After 3 d (or 5 d with Mu50 infection), mice were euthanized, and the catheters were removed aseptically and washed briefly with PBS. Catheters were placed in 1 ml of sterile PBS and sonicated to remove the adherent bacteria. The number of viable bacteria colonizing each catheter was determined by plating them on BHI agar plates with specific required antibiotics. Representative catheters removed from infected mice were fixed, sectioned, and subjected to further manipulation in the microscopy core laboratory at Chang Gung Memorial Hospital (CGMH). The biofilm bacteria were visualized under a scanning electron microscope (SEM) to evaluate the biofilm forming capacity under different treatments.

For the bladder catheter model, rats were anesthetized with 1.25% 2-2-2 tribromoethanol as above. A 0.5-cm segment of size Fr-10 silicon catheter was implanted into the urinary bladder, and the wound was closed with surgical glue. This was followed by the injection of 10^3^ CFU of *S*. *aureus* in a total volume of 100 μl of PBS into the bladder. After 1 h, vancomycin (40 mg/kg/dose) or control PBS buffer was administered twice daily by intravenous injection. After 3 d, the rats were euthanized, and the catheters were removed as described above.

### Detection of PIA

PIA was extracted from cells cultured in petri dishes according to an immuno-chemiluminescence method described previously, with minor modifications [20]. The culture conditions were similar to those used in the static biofilm adherence assay. Briefly, PIA was blotted onto a PVDF membrane (Millipore, Billerica, USA) using a 96-well dot-blot apparatus. The membrane was then dried and soaked in a solution containing 3% bovine serum albumin and 0.05% Tween-20 in PBS followed by incubation for 1 h at room temperature in a solution containing 0.8 μg/ml wheat germ agglutinin conjugated with biotin (WGA-biotin; Sigma-Aldrich). After three washes with PBS, PIA was detected using horseradish peroxidase-conjugated streptavidin (Pierce, Rockford, USA) followed by chemiluminescence detection.

### Real-time quantitative reverse-transcription PCR

Overnight cultures of strains SJC1200 were diluted to an OD_600_ of 0.1 with fresh BHI broth and grown at 37°C with shaking. Vancomycin (32 μg/ml), glucose [0.5% (BHIg) or 1.5% (BHIhg), w/v] or both was added to the bacterial cultures immediately after the glucose in original BHI was exhausted (T_0_), and bacterial cells were collected and pelleted after 1, 2, and 3 h (T1, T2, and T3, respectively) and frozen on dry ice immediately. Total RNA was extracted from cell pellets using the TRIzol (Invitrogen, Carlsbad, USA) method followed by RQ1 RNase-free DNase (Promega, Madison, USA) treatment to eliminate any remaining DNA. mRNA levels were determined by real-time quantitative reverse transcription PCR (qRT-PCR) with the KAPA SYBR qPCR Kit (Kapa Biosystems, Wilmington, USA) in a Roche LightCycler (LC-32; Roche). The primers used for *cidA* amplification were described previously [5]. Amplification of *agrA* was performed by primers agrA-F: 5’-TCGTAAGCATGACCCAGTTG-3’ and agrA-R: 5’-CACCGATGCATAGCAGTGTT-3’. All of the samples were tested in triplicate in three independent experiments. The expression levels of genes were normalized against the *dnaA* expression level. The fold change of each transcript was determined by the 2^−ΔΔCT^ method and compared with untreated cells [22].

### Nuclease activity assay

The supernatant (10 μl) from cultures in a static biofilm assay was incubated with 2 mg of PCR-amplified *hla* DNA product (861 bp) in a total volume of 100 μl at 37°C for 2 h. A positive control was performed by incubating the PCR product with 2 units of DNaseI (Promega). All of the reactions were stopped by the addition of 10 μl of EGTA (20 mM; Promega) and were then incubated at 65°C for 10 min; DNA degradation was then visualized using agarose gel electrophoresis and ethidium bromide staining and quantified using an image processing system.

### Statistical analysis

Student’s *t*-test was used to analyze experimental data and to compare the means. *P* values of less than 0.05 were considered statistically significant.

## Results

### Glucose availability determines the role of vancomycin in VRSA biofilm formation

**Fig 1:**
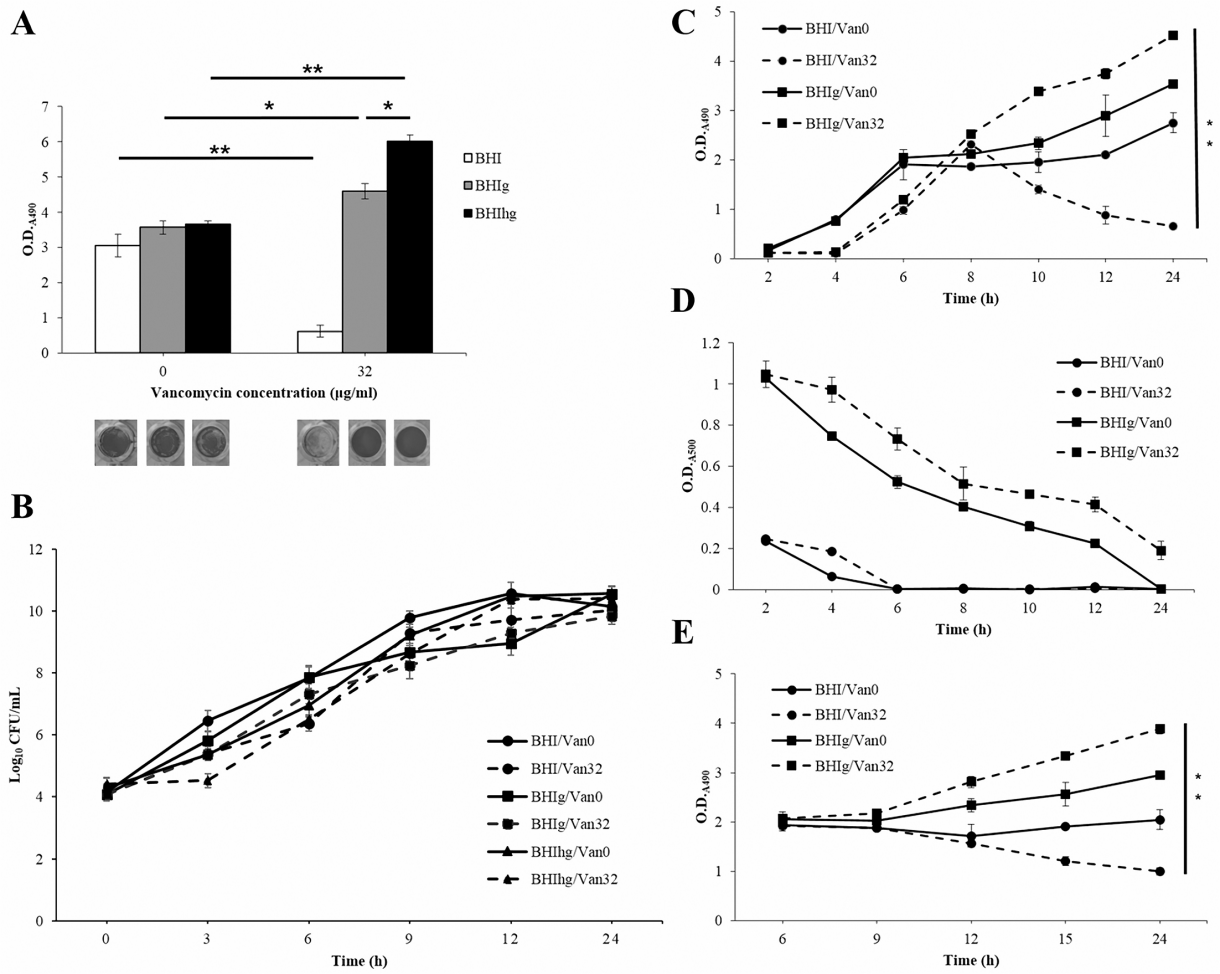
Vancomycin enhances biofilm formation with glucose but reduces propensity to form biofilms without glucose. (A) A static biofilm assay was performed when VRSA cells (SJC1200) were cultured in BHI medium or medium supplemented with 0.5% (BHIg) or 1.5% (BHIhg) glucose, respectively, in the absence/presence of vancomycin (32 μg/ml). Adherent cells from representative triplicate assays in each condition were stained with safranin O and are shown on the bottom. (B) A time course of the number viable cells was performed when VRSA cells were cultured in BHI or BHIg in the absence (Van0) or presence of vancomycin (32 μg/ml; Van32). The results are presented as the means±sd of the log_10_ CFU/ml from three separate experiments. (C) A time course of the static biofilm assay was performed when VRSA cells were cultured in different conditions as above. (D) The time course of glucose consumption. (E) A time course of the static biofilm assay was performed as above, but vancomycin was added immediately after the glucose was exhausted in BHI (at 6 h). * *P* < 0.05 and ** *P* < 0.005 in this figure and hereafter.

The static staphylococcal biofilm assay is always performed in BHI medium (containing 0.2% glucose) or other media, in which an additional 0.5% glucose (BHIg) was added [20]. We have previously demonstrated vancomycin-enhanced biofilm formation of the VRSA strain SJC1200 through a bacterial autolysis mechanism when bacteria were cultured in BHIg broth [5]. PIA, composed of the glucose metabolic derivative β-1,6-linked N-acetylglucosamin, is the major biofilm extracellular polymeric substance [23]. We then investigated the effect of the glucose concentration on vancomycin-enhanced biofilm formation. A slight but not significant increase in biofilm materials was observed when bacterial cells were cultured in BHIg and BHIhg compared to BHI broth (Fig 1A). Vancomycin significantly enhanced biofilm formation in BHIg, and the effect was even stronger in BHIhg broth (*P* < 0.005). Interestingly, biofilm formation was significantly suppressed in BHI broth in the presence of vancomycin (*P* < 0.005; Fig 1A). Similar results were observed when VISA strain Mu50 was employed under the same conditions (S1 Fig). To rule out the possibility that the difference in the amount of biofilm materials was due to the variation of bacterial viability among different culture conditions, time courses of the number of viable cells (CFU/ml) were performed. Slower bacterial growth was observed in the presence compared to the absence of vancomycin for the first 9 hours, and no significant difference was observed for the duration of the experiment (24 h; Fig 1B).

To determine whether reduced biofilm formation was due to the inhibition of biofilm development or the degradation of the formed biofilms in BHI with vancomycin, time courses of the static biofilm assay were performed. The results shown in Fig 1C indicate that the rate of production of biofilm materials was similar among all culture conditions during the exponential growth phase. Vancomycin significantly enhanced biofilm formation in BHIg throughout the duration of the experiment. A slower increase in biofilm materials was observed in vancomycin-free BHIg, whereas the amount of biofilm materials was kept at a relatively stable level 6 h post-inoculation when BHI was used. However, the formed biofilms were apparently degraded 6 to 8 h post-inoculation in BHI in the presence of vancomycin (Fig 1C). The four distinct modes of biofilm development, discussed above, were thought to be due to the availability of environmental glucose. Therefore, time courses of glucose consumption under the four culture conditions were performed. Approximately 6 h post-inoculation, glucose was exhausted in BHI with and without vancomycin (Fig 1D). Next, a time course of the static biofilm assay was also performed but vancomycin was added to the medium immediately after the glucose was exhausted in BHI, at 6 h post-inoculation. Similar to the results shown in Fig 1C, the availability of glucose determined the role of vancomycin in biofilm formation (Fig 1E).

### Vancomycin significantly enhances catheter-associated biofilm formation in diabetic mice infected by VRSA

**Fig 2:**
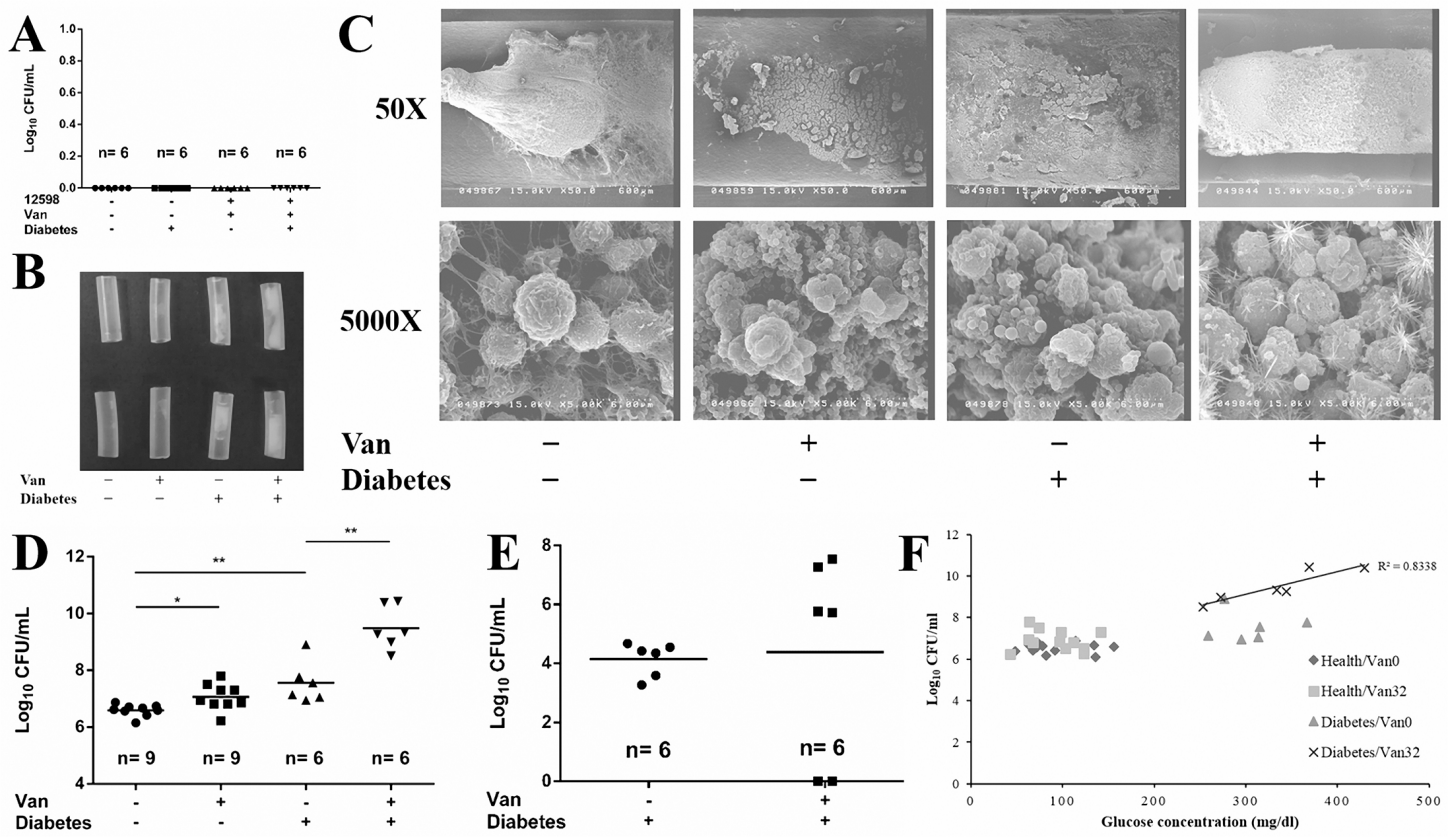
Vancomycin enhances catheter-associated biofilm formation in diabetic mice infected with vancomycin-non-susceptible *S*. *aureus*. (A) Experimental control of catheter-associated biofilm formation assay in mice. Determination of the number of viable biofilm-bound bacteria in subcutaneous catheters removed from healthy/diabetic mice upon infection with VSSA strain ATCC 12598 in the presence of vancomycin treatment. (B) Subcutaneous catheters removed from healthy/diabetic mice upon infection with VRSA strain SJC1200 in the absence/presence of vancomycin treatment. Two representative catheters in each group are shown in the figure. (C) Catheter-associated biofilm materials were observed under SEM. Photos were taken at the indicated magnifications. (D) Determination of the number of viable biofilm-bound bacteria in the removed catheters, as above. (E) Determination of the number of viable biofilm-bound bacteria in the catheters removed from diabetic mice infected with VISA strain Mu50 following vancomycin treatment. (F) The correlation between the blood glucose concentration and catheter biofilm-forming capacity. The capacity was evaluated by determining the number of viable biofilm-bound bacteria in subcutaneous catheters. The number of mice used in each group is shown.

To assess whether an increased blood or tissue glucose concentration reinforce vancomycin-enhanced biofilm formation in diabetic mice over the levels in healthy mice, a subcutaneously implanted catheter model was used. There were four control groups in this experiment: catheter lumens were injected with PBS in diabetic/healthy mice to confirm the aseptic manipulation; lumens were inoculated with vancomycin susceptible strain ATCC 12598 in diabetic/healthy mice, followed by vancomycin treatment to assess the effectiveness of the vancomycin concentration used in treating infections with drug-susceptible strains. Bacterial colonies were not found in any of the groups (Fig 2A). There was no difference of the *in vitro* biofilm forming capacity between strain 12598 and its derived VRSA strain SJC1200 (S2 Fig). The healthy/diabetic mice were then infected by VRSA with/without vancomycin treatment. The results shown in Fig 2B indicate that a thin layer of biofilm materials was observed on the inner wall of the catheters removed from healthy mice regardless of vancomycin treatment. A clot of biofilm materials was formed in the lumen of the catheters that were removed from vancomycin-untreated diabetic mice. However, the lumen was full of biofilm materials in the catheters from vancomycin-treated diabetic mice. The catheter-associated biofilms were then observed under SEM, and the lumen of the catheter from vancomycin-treated diabetic mice was full of a special spherical form of biofilm materials that were gathered together (Fig 2C).

The biofilm-forming capacity was evaluated by determining the biofilm-bound CFUs. A slight but significant increase (3-fold; *P* < 0.05) in the CFUs was detected in vancomycin-treated compared to untreated healthy mice (Fig 2D). A nearly 10-fold increase (*P* < 0.005) in the average CFU was observed in samples from diabetic mice without vancomycin treatment compared with vancomycin-untreated healthy mice. Amazingly, the average CFU was 1000-fold higher (*P* < 0.0001) in samples from vancomycin-treated diabetic mice than that from untreated healthy mice (Fig 2D). Experiments were repeated in diabetic mice, but the VRSA strain was replaced by VISA strain Mu50. Upon vancomycin treatment, Mu50 was cleared in two of the six mice. However, the biofilm forming capacity in the remaining four was significantly higher than that in vancomycin-untreated mice (Fig 2E). In addition, a high-positive correlation (R^2^ = 0.83) between the biofilm-forming capacity and blood glucose concentration in diabetic mice upon vancomycin treatment was observed, whereas there was no obvious correlation in the absence of vancomycin (Fig 2F). Therefore, sub-lethal levels of vancomycin treatment significantly enhanced boifilm formation in diabetic mice infected with vancomycin-non-susceptible *S*. *aureus* strains.

### Bacterial autolysis plays an important role in vancomycin-enhanced biofilm formation in diabetic mice

**Fig 3:**
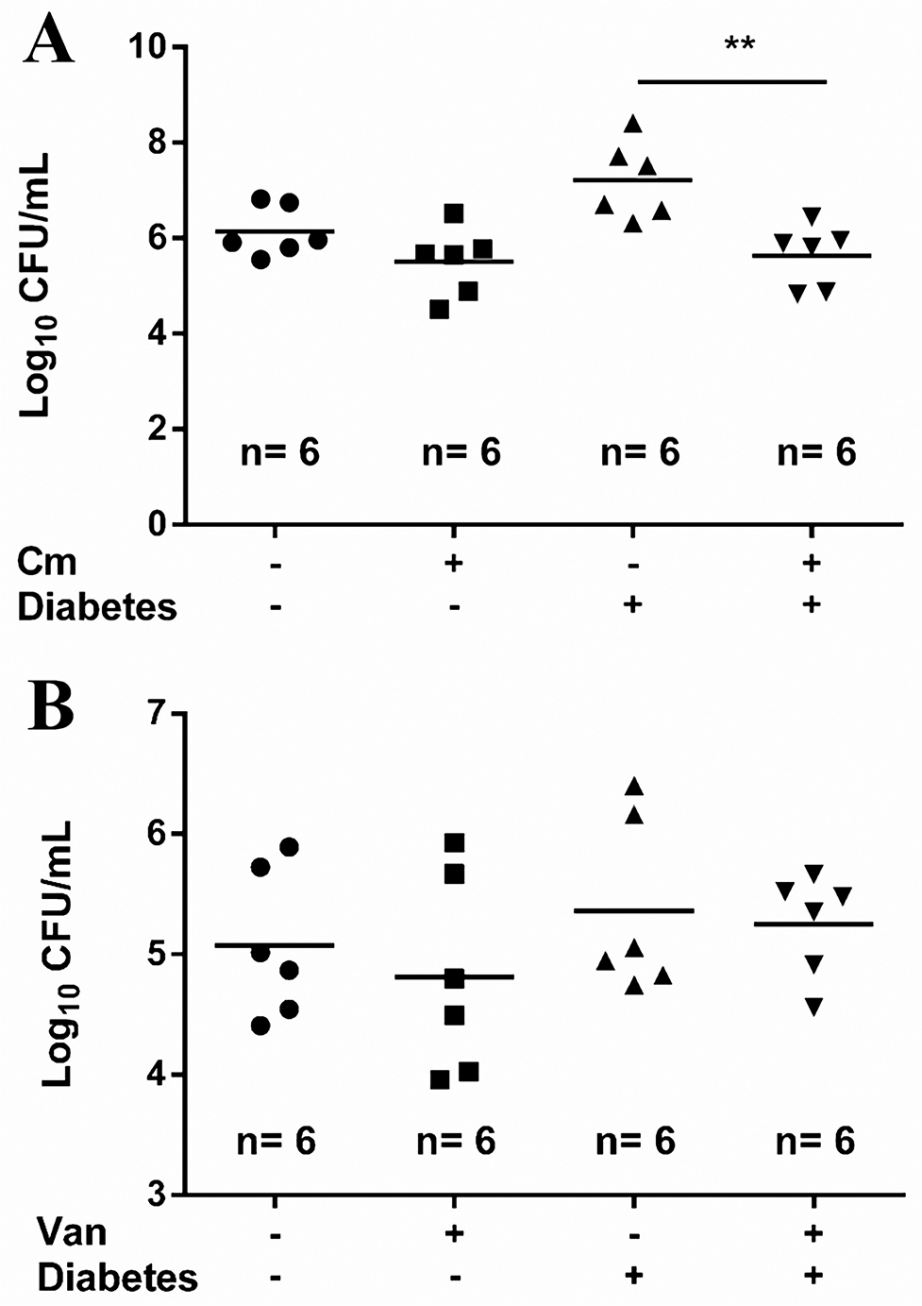
Bacterial autolysis triggered by vancomycin plays an important role in the enhancement of biofilm formation. Determination of the number of viable biofilm-bound bacteria in subcutaneous catheters removed from healthy/diabetic mice (A) infected with VRSA strain SJC1200 (also chloramphenicol resistance) upon chloramphenicol (Cm) treatment or (B) infected with *cidA* null mutant SJC1201 (autolysis deficient VRSA) upon vancomycin treatment. Six mice were used in each group.

We previously demonstrated that cell wall-active antibiotics but protein-suppressing agents triggered the *cidA*-mediated autolysis of *S*. *aureus*, leading to the enhancement of biofilm formation *in vitro*. Such enhancement was blocked when a *cidA* knockout VRSA strain SJC1201 was used [5]. Because pG1546 in SJC1200 also carries a chloramphenicol resistance cassette, vancomycin treatment was replaced by chloramphenicol, as described in the methods section. The results shown in Fig 3A indicate that chloramphenicol treatment for SJC1200 slightly reduced biofilm formation in healthy mice, but the reduction was significant (*P* < 0.0001) in diabetic mice. In addition, a similar level of biofilm-bound CFUs was observed when healthy/diabetic mice were infected by SJC1201 with/without vancomycin treatment (Fig 3B). The infected chloramphenicol susceptible strain, ATCC 12598, was efficaciously cleared in mice with the drug concentration used in this experiment (data not shown). As a result, release of eDNA triggered by cell wall-active antibiotics played a role in the enhancement of biofilm formation of *S*. *aureus* in diabetic mice.

### Vancomycin plays distinct roles in bladder catheter biofilm formation between healthy and diabetic rats upon VRSA infection

**Fig 4:**
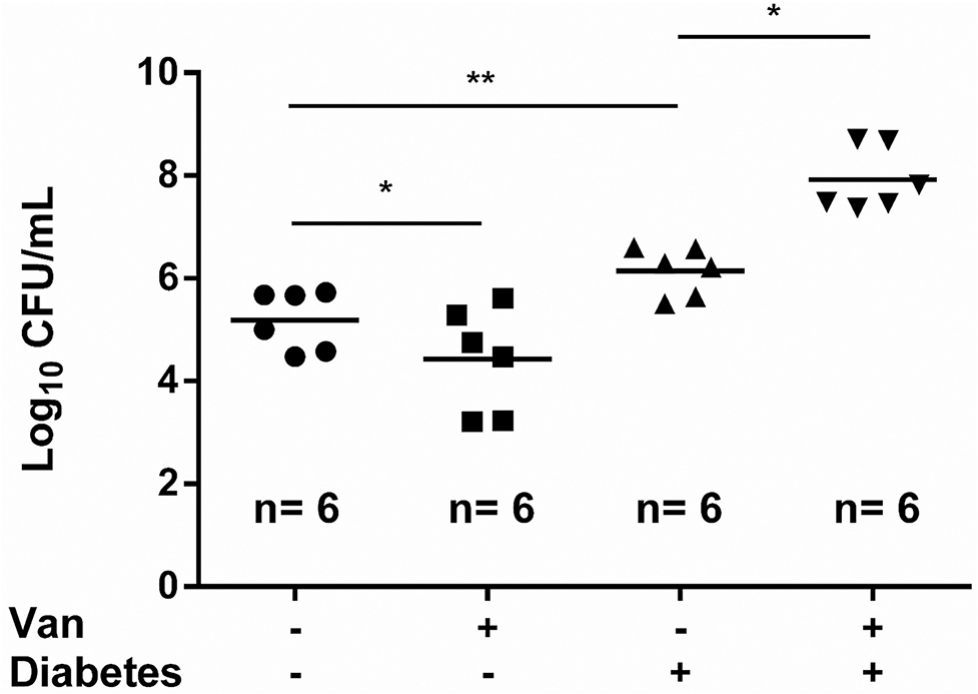
Vancomycin plays a different role in bladder catheter biofilm formation between healthy and diabetic rats. Determination of the number of viable biofilm-bound bacteria in the bladder catheters removed from healthy/diabetic rats infected with VRSA SJC1200 in the absence/presence of vancomycin treatment. Six rats were used in each group.

As shown in Fig 1, vancomycin reduced the propensity to form biofilms in the absence of glucose, and the urine glucose concentration is always undetectable in healthy people and rats. We therefore investigated the effect of vancomycin treatment on catheter-associated biofilm formation in a urinary bladder following the same treatment protocol as above. Mice were replaced by rats because of the enough bladder space for the implantation of catheters. Both the inner and outer walls of the catheter were covered by biofilm materials when the catheters were removed from the bladders. Among the healthy rats infected by VRSA, a significant decrease in biofilm formation was found in vancomycin-treated rats compared to untreated rats (*P* < 0.05). The biofilm-forming capacity is approximately 10 times higher in diabetic rats than that in healthy rats when vancomycin was not applied (*P* < 0.001). A further 10-fold increase in biofilm formation was observed in diabetic rats upon vancomycin treatment (*P* < 0.05; Fig 4). Above results suggested that glucose was essential for the vancomycin-enhanced biofilm formation.

### Mechanisms underlying biofilm degradation upon vancomycin treatment in the absence of glucose

**Fig 5:**
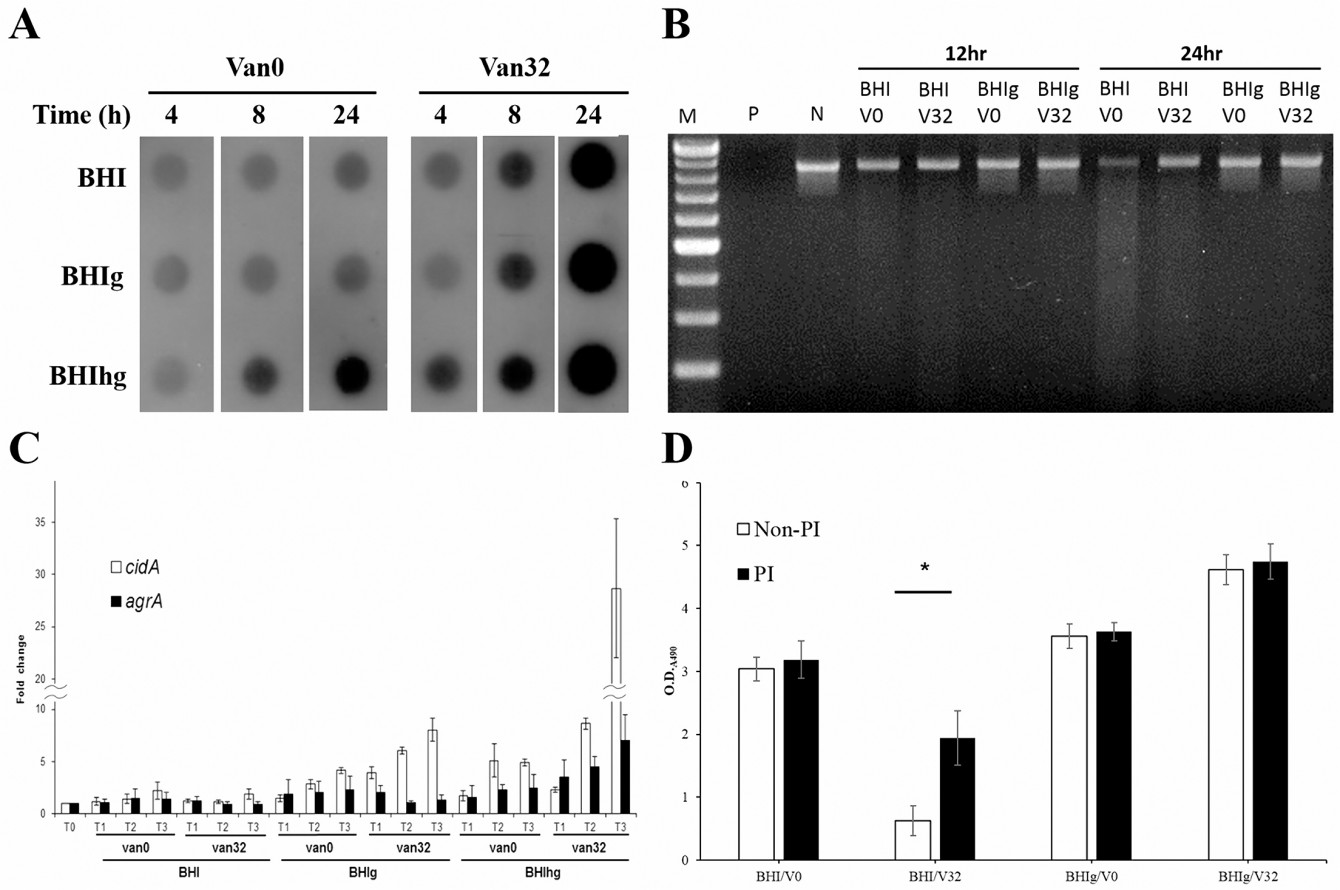
Investigation of the mechanisms underlying vancomycin-triggered biofilm degradation in the absence of glucose. (A) Time courses of PIA production by VRSA strain SJC1200 in different media with/without vancomycin treatment. (B) Evaluation of bacteria-secreted DNase activity by visualizing DNA degradation after incubating a DNA product (861 bp) with the supernatant removed from different VRSA culture conditions. 12 hr and 24 hr: supernatant from a 12-h and a 24-h culture system, respectively. M: a 100-bp DNA ladder marker. P and N: positive and negative controls, respectively. (C) Detection of changes in the transcription levels of *cidA* and *agrA* in VRSA by qRT-PCR upon different treatments. The fold change of each transcript was compared with vancomycin-untreated samples at T_0_. (D) Detection of the bacteria-secreted protease activity by the evaluation of the biofilm degradation (suppression) potential using a static biofilm assay in the absence (Non-PI) or presence (PI) of a protease inhibitor cocktail.

The expression level of selected major factors involved in matrix accumulation and degradation was measured for characterizing the vancomycin effect. In the absence of vancomycin, the expression of PIA was higher when bacterial cells were cultured in BHIhg than in the other two media. However, the expression of PIA was significantly stronger in the presence of vancomycin, regardless of the presence of glucose (Fig 5A). Therefore, expression of PIA was not the cause of biofilm degradation. The activity of extracellular nuclease was measured to evaluate the potential of a secreted nuclease for degrading biofilm-bound eDNA. A higher nuclease activity was observed in the supernatant removed from BHI, both in 12 h and 24 h cultures compared to the supernatant from BHIg, regardless of the presence of vancomycin. Among samples from 24 h cultures, a 77%, 36%, 16%, and 11% decrease in the DNA load was observed in BHI/V0, BHI/V32, BHIg/V0, and BHIg/V32, respectively (Fig 5B). This result suggests that the release of nuclease by *S*. *aureus* is enhanced in the absence of glucose whereas vancomycin may not contribute to the nuclease release. Release of eDNA was evaluated by measuring the transcriptional level of *cidA*. Consistent with previous findings, vancomycin significantly enhanced *cidA* expression when bacterial cells were cultured in BHIg, and the effect was even stronger in BHIhg [5]. However, there was no significant difference in *cidA* expression between vancomycin-treated and-untreated cells cultured in BHI at each of the time points (Fig 5C). This result implies that release of eDNA is suppressed in the absence of glucose regardless the presence of vancomycin. Different types of PSMs which are regulated by the global regulator Agr, have been reported to be involved in biofilm degradation [10]. Thus, expression levels of *agrA* were measured by qRT-PCR. A slight suppression of the expression level of *agrA* was detected in BHI and BHIg upon vancomycin treatment. However, a significant increase in *agrA* expression in BHIhg following vancomycin treatment at each time point was observed in comparison to the vancomycin-free counterparts (Fig 5C). The activity of proteases was evaluated by the addition of a protease inhibitor cocktail to the culture media. The inhibitors significantly reduced vancomycin-enhanced biofilm degradation capacity in BHI suggesting that vnacomycin promoted the expression of protease in the absence of glucose (Fig 5D).

## Discussion

In the present study, we demonstrate that a sub-lethal dose antibiotic treatment, especially with a cell wall active antibiotic, such as vancomycin, in diabetic mice infected with drug-resistant *S*. *aureus* strongly enhanced biofilm formation, possibly through *cidA*-mediated bacterial autolysis. A positive correlation between the blood glucose concentration and the amount of biofilm materials was observed when improper antibiotic treatment was applied. We also showed that the availability of environmental glucose determined the role of vancomycin in the biofilm formation of *S*. *aureus*. Since vancomycin is one of the frequent use antibiotics in treating MDRSA and has a longer half-life than oxacillin, infection with VISA or VRSA strains were used in our animal model.

Diabetes mellitus has long been recognized as one of the major risk factors in bacteria-associated infections, such as periodontal diseases, pneumonia, urinary tract infections, blood stream infections, among others [24–27]. Patients with diabetes sometimes suffer from chronic infections, particularly in their feet. The development of diabetic foot infections is supposedly due to local neuropathy, vascular changes and depressed local host defenses [28]. However, chronic infections as well as medical device-associated infections are also highly correlated with the formation of biofilms [14, 29]. Fewer published studies address mechanisms underlying biofilm formation in patients with diabetes. Therefore, to the best of our knowledge, no literatures describe the impact of improper antibiotic treatment on infection and biofilm formation caused by drug-resistant bacteria in a diabetic environment. A recent study indicates that among the 255 bacteria isolated from 162 patients with diabetic foot ulcers, 179 of them were biofilm producers [30]. It has been demonstrated that wound healing was delayed in diabetic mice following to biofilm challenge [15].

In this study, we demonstrate that the biofilm-forming capacity was approximately 10 times higher in diabetic mice than in healthy ones (Fig 2D). We suggest that this supports the role of a diabetic environment in enhancing biofilm formation, as reported in many clinical observations [15, 16]. Mechanisms underlying such enhancement may range from the host response, as described above, to bacterial factors, such as the response to environmental glucose, as has been addressed herein. We also show that vancomycin treatment at a therapeutic concentration efficaciously eliminated bacterial infections in both healthy and diabetic mice infected with drug-susceptible *S*. *aureus* strains (Fig 2A). However, even in healthy mice, sub-lethal doses or improper antibiotic treatment still significantly enhanced biofilm formation when mice were infected by drug-resistant *S*. *aureus* (Fig 2D).

A striking finding in this study is that a tremendous increase in catheter-associated biofilm materials was observed in diabetic mice infected either by VRSA or VISA upon vancomycin treatment (Fig 2). Following VISA infection, once the strain could tolerate the antibiotic pressure, the biofilm forming capacity was significantly higher than that in drug-untreated diabetic mice (Fig 2E). The mechanism underlying biofilm enhancement may reflect our previous findings that cell wall-active antibiotics and SXT trigger the release of eDNA through *cidA*-mediated bacterial autolysis, leading to the enhancement of biofilm formation *in vitro* [5]. This concern was supported when vancomycin was replaced by a protein synthesis suppressing agent (chloramphenicol) or when a *cidA* knock out VRSA strain was used in our animal model and antibiotic-triggered biofilm enhancement was abolished (Fig 3). Through this *in vitro* study, vancomycin reduced propensity to form biofilm in the absence of glucose, which promoted the investigation of the bladder catheter-associated biofilm-forming capacity between diabetic and healthy rats. As expected, a significant suppression in biofilm-forming capacity was observed in catheters removed from healthy rat bladders, even if they were infected with VRSA concomitant with vancomycin treatment (Fig 4). This result suggests that improper antibiotic treatment may not be a risk factor for biofilm formation in non-diabetic patients using urinary catheters. However, the diabetic environment itself apparently promoted bladder catheter biofilm formation, and this effect was even stronger with improper antibiotic treatment upon infection with drug-resistant *S*. *aureus* (Fig 4). Thus, prophylactic antibiotic treatment with cell wall-active agents or SXT in diabetic patients with a urinary tract infection (UTI) or who are using an indwelling urinary catheter should be considered. A recent study also indicated that patients with diabetes showed a significantly higher probability (4.55 times) of having a catheter-associated UTI than those without diabetes [31]. A previous study indicated that the highest mean steady-state concentrations of vancomycin in continuous and intermittent infusion regimens were 24.88±12.75 and 55.02±17.36 μg/ml, respectively, whereas the lowest concentrations were 19.89±10.15 and 12.43±12.86 μg/ml, respectively, in serum [32]. The dose of vancomycin administered in our mouse model allows the concentration of vancomycin in tissue fluid to maintain in a therapeutic level. However, the vancomycin concentration in rat urine was only 60% of that in mouse subcutaneous tissues (12 and 20 μg/ml, respectively) and was 20% lower in the diabetic environment when a single dose per day was applied in this study. Therefore, two doses of vancomycin were administered daily in the rat bladder catheter experiment.

Another interesting finding in this study is that the availability of glucose determined the role of vancomycin in biofilm formation of VRSA. PIA is the major matrix component in PIA-dependent biofilms, including the biofilm produced by the strain SJC1200 used in this study [5]. Thus far, Dispersin B (DspB) is the only enzyme that specifically hydrolyzes PIA, but it is only produced by *Actinobacillus actinomycetemcomitans* [33]. In addition, regardless of the presence of glucose, vancomycin promoted PIA production, suggesting that PIA synthesis may not play a role in vancomycin-enhanced biofilm degradation (Fig 5A). In the absence of glucose, DNase activity increased and the expression of *cidA* was suppressed regardless of the presence of vancomycin, implying that the release of eDNA was not the only factor responsible for the vancomycin-enhanced biofilm degradation (Fig 5B and 5C). Including MSCRAMMs, lots of proteins are associated with biofilm development, and the expression of each of them cannot be investigated [6]. The addition of a protease inhibitor cocktail to BHI/vancomycin suppressed approximately 50% of biofilm degradation, suggesting that the expression of more proteases in BHI/vancomycin may be one of the major contributors to biofilm degradation (Fig 5D). Expression of PSMs is strictly regulated by an *agr* quorum sensing system [34]. Although the expression of different PSMs was not measured in this study, the suppressed *agrA* expression upon vancomycin treatment in BHI and BHIg suggested that PSMs may not be centrally involved in biofilm degradation (Fig 5C). We previously demonstrated that σ^B^ in VRSA is activated upon vancomycin treatment in BHI [4]. It has been reported that *agr* RNAIII levels were elevated in the *sigB* mutants [35]. It is suggested that vancomycin treatment activates σ^B^, leading to the suppression of *agr* expression in VRSA.

In conclusion, our study provides a warning of the potential risks when cell wall-active antibiotics, such as vancomycin, are initially applied for treating infections caused by drug-resistant *S*. *aureus* or other biofilm producers in patients with diabetes. A previous study indicated that vancomycin tissue concentrations in diabetic patients were approximately 30% of those in nondiabetics [36]. As a result, bacteria with a lower MIC, such as VISA, can survive and produce large amounts of biofilms, leading to difficulties in subsequent treatments. Because we demonstrated a high association between glucose concentration and biofilm-forming capacity, the effect of insulin treatment on infection or biofilm formation in a diabetic environment is controversial. A recent study shows that insulin treatment improved the surgical site infection outcome by *S*. *aureus* in diabetic mice, potentially through the enhancement of neutrophil function [37]. However, another study demonstrates that insulin treatment resulted in an increase in lysed neutrophils along with fewer macrophages to remove cell debris, leading to an increase in DNA levels, thereby enhancing *Pseudomonas aeruginosa* wound biofilms in diabetic mice [38]. Nevertheless, the impact of insulin treatment on biofilm formation in our model system needs to be studied further.

## Supporting Information

S1 Fig Vancomycin enhances biofilm formation of VISA strain Mu50 with glucose. A static biofilm assay was performed when VISA cells (Mu50) were cultured in BHI medium or medium supplemented with 0.5% (BHIg) or 1.5% (BHIhg) glucose, respectively, in the absence/presence of vancomycin (4 μg/ml).
(TIF)

S2 Fig Comparison of biofilm forming capacity between *S*. *aureus* strains 12598 and SJC1200. A static biofilm assay was performed when *S*. *aureus* strains 12598 and SJC1200 were cultured in BHI, BHIg, or BHIhg, respectively.
(TIF)
