# Supplementary figures and images for: High Glucose Concentration Promotes Vancomycin-Enhanced Biofilm Formation of Vancomycin-Non-Susceptible Staphylococcus aureus in Diabetic Mice
Source: PLoS One. 2015 Aug 5;10(8):e0134852. doi: 10.1371/journal.pone.0134852 (PMC4526670; doi:10.1371/journal.pone.0134852)

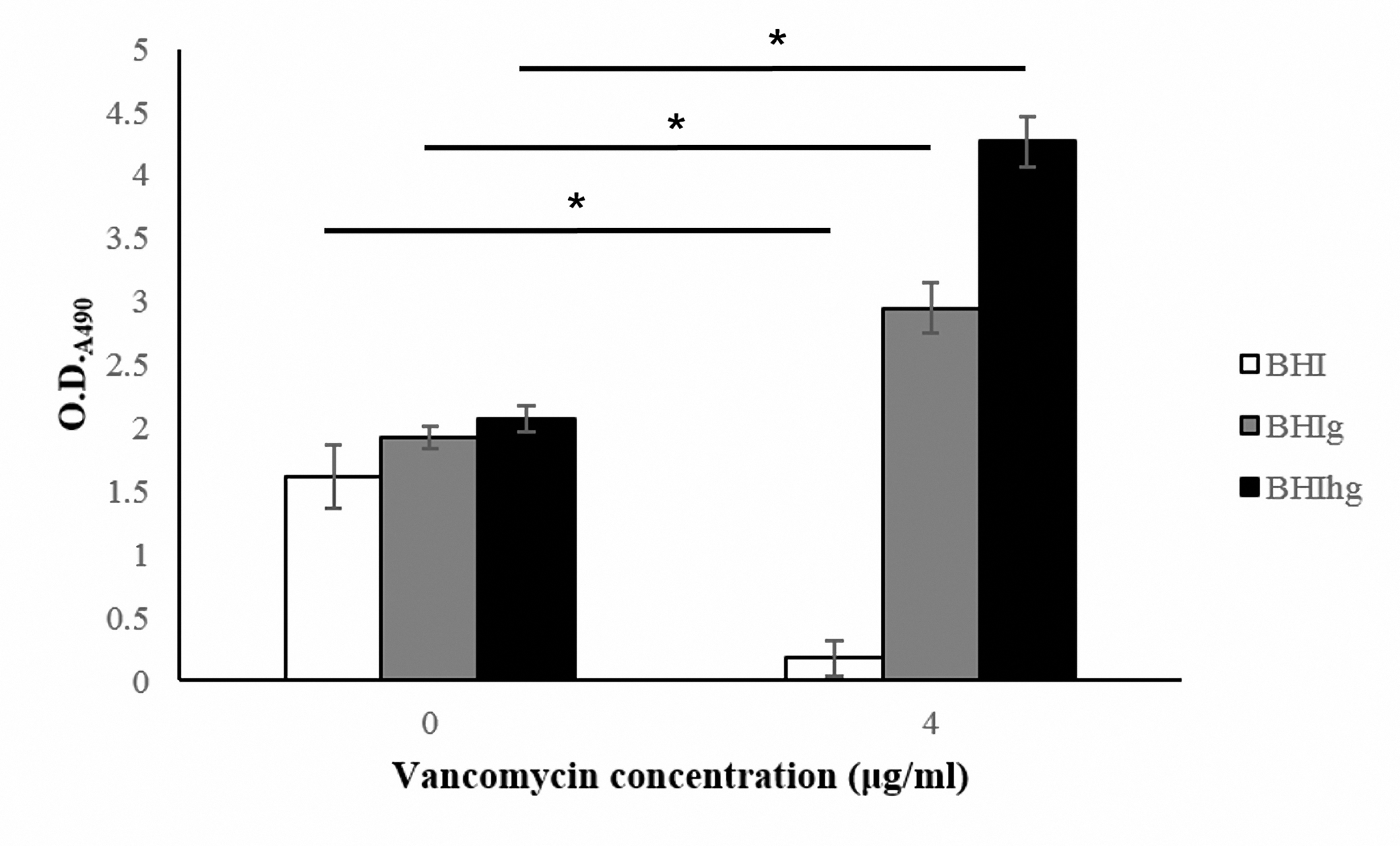

Supplement: S1 Fig — A static biofilm assay was performed when VISA cells (Mu50) were cultured in BHI medium or medium supplemented with 0.5% (BHIg) or 1.5% (BHIhg) glucose, respectively, in the absence/presence of vancomycin (4 μg/ml). (TIF) [file pone.0134852.s001.TIF]

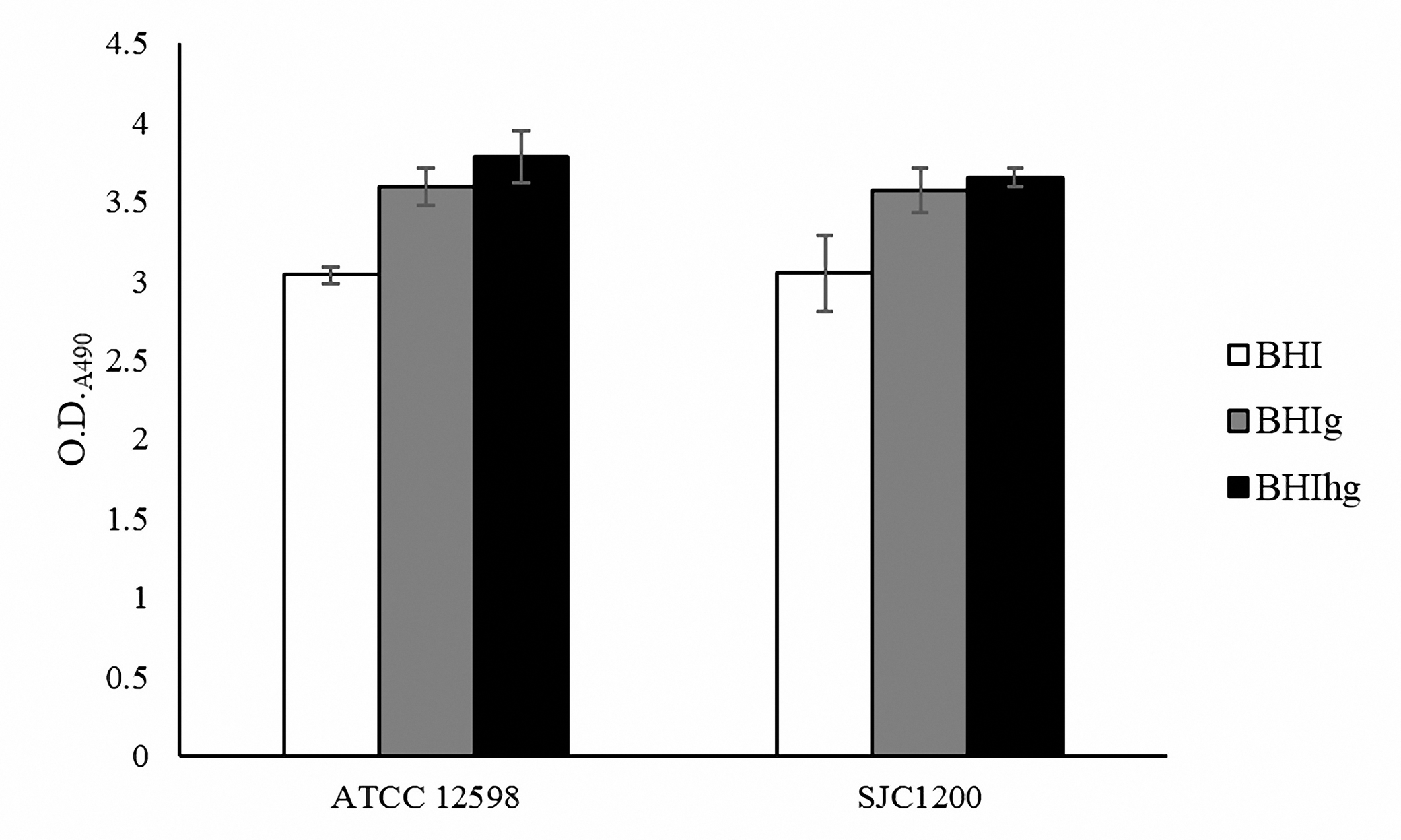

Supplement: S2 Fig — A static biofilm assay was performed when S. aureus strains 12598 and SJC1200 were cultured in BHI, BHIg, or BHIhg, respectively. (TIF) [file pone.0134852.s002.TIF]
